# α-Pinene Improves Follicle Morphology and Increases the Expression of mRNA for Nuclear Factor Erythroid 2-Related Factor 2 and Peroxiredoxin 6 in Bovine Ovarian Tissues Cultured In Vitro

**DOI:** 10.3390/ani14101443

**Published:** 2024-05-12

**Authors:** Venância Antonia Nunes Azevedo, Ernando Igo Teixeira De Assis, Anderson Weiny Barbalho Silva, Francisco Das Chagas Costa, Layana Freitas Souza, José Roberto Viana Silva

**Affiliations:** 1 Laboratory of Biotechnology and Physiology of Reproduction, Federal University of Ceara, Sobral 62041-040, CE, Brazil; 2 Laboratory of Biochemistry and Gene Expression, State University of Ceara, Fortaleza 60714-903, CE, Brazil

**Keywords:** preantral follicles, antioxidant, morphology, cow, ovarian stroma

## Abstract

**Simple Summary:**

The oxidative stress during in vitro culture of ovarian tissues can reduce the production of collagen fibers in the extracellular matrix and impair follicular and stromal cell survival. Consequently, there is a necessity to supplement culture media with antioxidant agents to minimize the damage caused by oxidative stress. α-pinene is a natural monoterpene with a wide range of biological effects, including antioxidant activity. However, it is unclear whether it improves the efficiency of in vitro culture of bovine ovarian tissue. Therefore, we studied the effect of α-pinene on follicular activation, growth, and morphology, on stromal cell density and collagen fibers, as well as on the expression of genes involved in antioxidant defense in bovine ovarian tissues cultured in vitro. The results indicate that the presence of α-pinene in the culture medium improved follicular morphology but did not affect follicular growth. It preserves the density of stromal cells and collagen fibers and increases transcript levels of enzymes involved with antioxidant defense. In conclusion, α-pinene is a promising antioxidant substance, enhancing follicular and stromal cell quality and relieving oxidative stress.

**Abstract:**

Oxidative stress during in vitro of ovarian tissues has adverse effects on follicle survival. α-pinene is a monoterpenoid molecule with antioxidant activity that has great potential to maintain cell survival in vitro. This study investigated the effect of α-pinene (1.25, 2.5, 5.0, 10.0, or 20.0 μg/mL) on primordial follicle growth and morphology, as well as on stromal cells and collagen fibers in bovine ovarian slices cultured for six days. The effect of α-pinene on transcripts of catalase (*CAT*), superoxide dismutase (*SOD*), peroxiredoxin 6 (*PRDX6*), glutathione peroxidase (*GPX1*), and nuclear factor erythroid 2-related factor 2 (*NRF2*) was investigated by real-time PCR. The tissues were processed for histological analysis to evaluate follicular growth, morphology, stromal cell density, and collagen fibers. The results showed that 2.5, 5.0, or 10.0 µg/mL α-pinene increased the percentages of normal follicles but did not influence follicular growth. The α-pinene (10.0 µg/mL) kept the stromal cell density and collagen levels in cultured bovine ovarian tissue like uncultured tissues. Ovarian tissues cultured in control medium had reduced expression of mRNA for *NRF2*, *SOD*, *CAT*, *GPX1,* and *PRDX6*, but α-pinene (10.0 µg/mL) increased mRNA levels for *NRF2* and *PRDX6*. In conclusion, 10.0 µg/mL α-pinene improves the follicular survival, preserves stromal cell density and collagen levels, and increases transcripts of *NRF2* and *PRDX6* after in vitro culture of bovine ovarian tissue.

## 1. Introduction

The in vitro culture of ovarian tissue provides a complex support system that resembles the intraovarian physiological environment, where the follicle maintains contact with stromal cells and the extracellular matrix (ECM) [1]. In addition to providing structural support, bidirectional paracrine signaling among ovarian follicles and surrounding tissue can control follicular survival, activation, and growth [2]. Collagen, elastin, fibronectin, and laminin are compounds present in the ECM that form a structurally stable structure that works as a reservoir of hormones and growth factors, regulating their diffusion and availability within the ovarian environment [3]. Despite advances in understanding the control of early follicular development, culture of ovarian tissues is still associated with high production of reactive oxygen species (ROS), which trigger oxidative stress [4] and, consequently, result in high follicular degeneration [5,6,7].

The cellular antioxidant defense system in vitro is influenced by various factors, such as high oxygen concentrations, and the follicles are exposed to an excess of ROS that can cause mitochondrial and DNA damage, peroxidation of membrane phospholipids, and abnormal gene expression patterns [8]. ROS can disturb ECM homeostasis by changing the expression and activity of matrix metalloproteinases (MMPs) that are involved in the remodeling and degradation of ECM proteins, including collagen [9]. The oxidative stress also changes the expression of enzymes involved in antioxidant defense, i.e., catalase (*CAT*), superoxide dismutase (*SOD*), glutathione peroxidase (*GPX*), and peroxiredoxins (*PRDX*) [10]. Over the last few years, a promising alternative to prevent the in vitro harmful effects of ROS has been the use of natural antioxidants as supplements for culture medium.

α-pinene (C_10_H_16_) is a natural bicyclic monoterpene with a wide range of biological activities, such as anticoagulant, anti-inflammatory, antimicrobial, antitumor, and antioxidant [11]. Porres-Martinez et al. [12] reported that, in vitro, α-pinene protects U373-MG cells against the damages caused by H_2_O_2_ oxidative stress by maintaining morphology, cellular viability, and increasing glutathione, glutathione reductase, *GPX*, *CAT*, and *SOD* activities. The α-pinene also induced alterations in transcript levels of oxidative response-related genes, like nuclear factor erythroid 2-related factor 2 (*NRF2),* in HaCaT cells [13].

The aim of the present work was to investigate the effects of α-pinene, at different concentrations, on follicular activation, growth, morphology, ovarian stromal cell density, and the distribution of collagen fibers in the ECM of cultured bovine ovarian tissues. The influence of α-pinene on the expression of mRNA of antioxidant enzymes (*SOD*, *CAT*, *PRDX6*, *GPX1*) and *NRF2* in in vitro cultured ovarian cortex was also investigated.

## 2. Material and Methods

### 2.1. Source of Ovaries

The culture media, α-pinene, and other chemicals used in the study were purchased from Sigma Chemical Co., Ltd. (St. Louis, MO, USA), unless mentioned otherwise. 

Cow ovaries (*n* = 10 pairs) were collected at a local slaughterhouse. The ovaries were washed with 70% ethanol for approximately 10 s, followed by two rinses in a 0.9% saline solution. Then, the ovaries were transported to the laboratory in Minimum Essential Medium (MEM) supplemented with streptomycin (100 µg/mL) and penicillin (100 µg/mL) at 4 °C for a period of 1 h. This research was performed after the approval of ethics and Animal Welfare Committee of the Federal University of Ceará (N° 15/2021).

### 2.2. In Vitro Culture of Ovarian Tissue

In the laboratory, the ovarian cortical tissues of each cow (*n* = 10) were cut into 38 fragments (3 mm × 3 mm × 1 mm) in α-MEM supplemented with streptomycin (100 μg/mL) and penicillin (100 μg/mL). For each cow, cortical tissues were fixed in neutral buffered formaldehyde (10%) for 24 h at 4 °C to evaluate follicle morphology, stromal cell density, and distribution of collagen fibers in the ECM by classical histology. Cortical slices were also stored at −80 °C to evaluate the gene expression of mRNA (*CAT*, *SOD*, *GPX1*, *PRDX6*, and *NRF2*). The other fragments were cultured in 24-well culture dishes for 6 days at 38.5 °C in 5% CO_2_ in a humidified incubator [6]. The α-MEM (pH 7.2–7.4) with antibiotics (100 µg/mL streptomycin and 100 µg/mL penicillin), ITS (10 μg/mL insulin, 5.5 μg/mL transferrin, and 5 ng/mL selenium), 2 mM glutamine, 2 mM hypoxanthine, and 1.25 mg/mL of bovine serum albumin was the control culture medium. The tissues were cultured in 500 μL of control medium (α-MEM) alone or supplemented with α-pinene in different concentrations (1.25; 2.5; 5.0; 10.0; and 20.0 μg/mL). The concentrations of α-pinene were chosen according to Xanthis et al. [13]. Every two days, half of the culture medium was refreshed. After a six-day culture period, the ovarian slices were used for analysis of tissue morphology and gene expression. This experiment was repeated 10 times.

### 2.3. Evaluation of Follicle Growth and Survival 

Histological evaluation was performed according to a previous study [6]. The preantral follicles with oocyte nuclei visible in the sections were analyzed. The developmental stages of follicles were classified as primordial or growing follicles, i.e., primary or secondary, according to Figueiredo and Lima [14]. These follicles were further classified as morphologically health when had an intact oocyte and well-organized granulosa cells or as degenerated follicles that had retracted oocyte or disorganized granulosa cells detached from the basement membrane [15]. In general, 200–290 follicles were analyzed in each treatment. The rate of healthy primordial and developing follicles was reported before and after culture in a particular medium.

### 2.4. Evaluation of Stromal Cell Density and Collagen 

The density of stromal cells in uncultured and cultured ovarian tissues was determined by counting the stromal cell number in an area of 100 µm^2^, according to Cavalcante et al. [5]. The analysis of collagen fibers in the ovarian ECM was performed by Picrosirius red staining (Abcam Kit, Cambridge, UK), according to Rittié [16]. For each experimental condition, the area of collagen fibers in 20 different fields was evaluated with the aid of a camera coupled to a microscope (Nikon, Eclipse, TS 100, Tokyo, Japan). The collagen fibers stained red, while the follicles remained unstained. Quantification of collagen fibers in uncultured and cultured tissues was performed by ImageJ software (Version 1.51p, 2017). The area of red-marked collagen fibers was evaluated by measuring the pixel intensity of the total area after background subtraction.

### 2.5. Expression of mRNA for SOD, CAT, PRDX6, GPX1 and NRF2 

The samples were stored at −80 °C until the extraction of total RNA. Based on the results of follicular morphology, collagen fibers, and stromal cell density, ovarian tissues cultured in control medium alone or supplemented with 10.0 µg/mL α-pinene were selected to investigate mRNA expression. Following the manufacturer’s instructions, total RNA was extracted using a Trizol^®^ purification kit (Invitrogen, São Paulo, Brazil). To this, 800 µL of Trizol^®^ solution was added to each frozen sample, and the lysate was aspirated through a 20-gauge needle before centrifugation at 10,000 g for 3 min at room temperature. Thereafter, all lysates were diluted 1:1 with 70% ethanol and applied to a minicolumn provided in the kit. After binding of the RNA to the column, DNA digestion was performed using RNAse-free DNAse (340 K units/mL) for 15 min at room temperature. After washing the column three times, the RNA was eluted with 30 µL of RNAse-free water. The RNA samples were evaluated for their quality and quantity in a spectrophotometer (BioDrop, Cambridge, England) by reading the absorbance at 260 nm and purity checking at 280 nm. For each sample of ovarian tissue, 2.44 μg of total RNA was used for first-strand cDNA synthesis. The samples of RNA were incubated at 70 °C for 5 min and then cooled on ice. Reverse transcription was performed in a total volume of 20 μL composed of 4 μL reverse transcriptase buffer (Invitrogen), 10 μL of RNA sample, eight units of RNAse, 150 units of reverse transcriptase (Superscript III, Invitrogen, São Paulo, Brazil), 10 mM dithiothreitol, 0.036 U random primers, and 0.5 mM of each dNTP (Invitrogen). Then, the tubes were incubated for 1 h at 42.1 °C, subsequently for 5 min at 80 °C, and finally stored at −20 °C. The negative control did not have the addition of reverse transcriptase.

The real-time reactions were performed in a Step One Plus instrument (Applied Biosystems, Foster City, CA, USA) and contained 10 μL of SYBR Green Master Mix (Applied Biosystems, Warrington, UK), 7.3 μL of ultrapure water, 1 μL of complementary DNA (cDNA), and 5 mM of each primer. The primers were developed to specifically amplify *SOD*, *CAT*, *PRDX6*, *GPX1*, *NRF2*, and glyceraldehyde3-phosphate dehydrogenase (*GAPDH*) (Table 1). *GAPDH* was used as a housekeeping gene. The melting curve analysis of PCR products was used to confirm the specificity of each primer pair. The methodology previously described by Pfaffl et al. [17] was used to evaluate efficiency amplification for all genes. The cycling profile for the first round of PCR was denaturation and activation of the polymerase for 10 min at 95 °C, followed by 40 cycles of 15 s at 95 °C, 30 s at 58 °C, and 30 s at 72 °C. The final extension was for 10 min at 72 °C. The negative control was performed under the same conditions but without the addition of cDNA. The 2^∆∆Ct^ method was used to transform the C_t_ values into mRNA expression levels [18].

### 2.6. Statistical Analysis

A chi-square test was used to analyze the percentage of normal follicles and those of primordial and developing follicles in each treatment (GraphPad Prism 9.0). Data on stromal cell density and collagen fibers were analyzed by analysis of variance (ANOVA) and the Tukey test. The association between stromal cell density and the percentage of normal preantral follicles was evaluated by linear regression analysis. An unpaired Student’s *t*-test was used to compare the levels of mRNA, and the differences were statistically significant at *p* < 0.05. 

## 3. Results

### 3.1. Effects of α-Pinene on Follicular Morphology 

**Figure 1:**
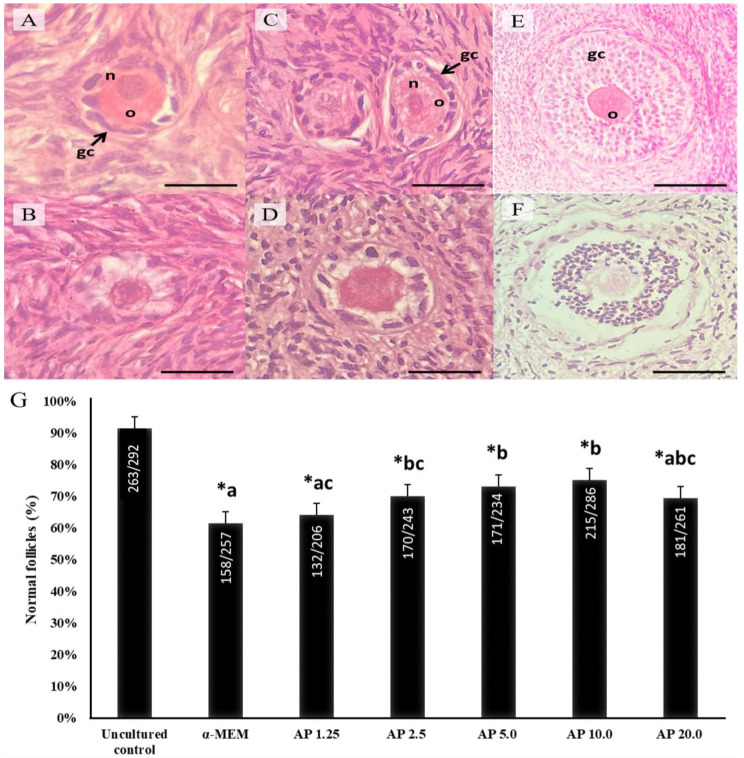
Morphology (**A**–**F**) and percentages (**G**) of normal follicles in uncultured samples and in samples cultured for 6 days in control medium alone (α-MEM) or with 1.25, 2.5, 5.0, 10.0 or 20.0 µg/mL α-pinene (AP). Normal and atretic primordial (**A**,**B**), primary (**C**,**D**) and secondary follicles (**E**,**F**). Granulosa cells (gc); oocyte (o); oocyte nucleus (n). Scale bar: 100 μm. a–c: statistically significant differences between treatments. * Differs significantly from uncultured tissues. Numbers of normal and total follicles evaluated are shown within each column.

Figure 1 illustrates normal and degenerate follicles in ovarian tissue samples before and after culture. After 6 days, cultured ovarian tissues had a decrease in the rate of normal follicles in all treatments when compared with uncultured tissues (*p* < 0.05). Ovarian tissues cultured with 2.5, 5.0, and 10.0 µg/mL α-pinene showed, however, a higher rate of normal follicles than those cultured in α-MEM alone (*p* < 0.05) (Figure 1).

### 3.2. Effects of α-Pinene on Activation and Development of Primordial Follicles 

**Figure 2:**
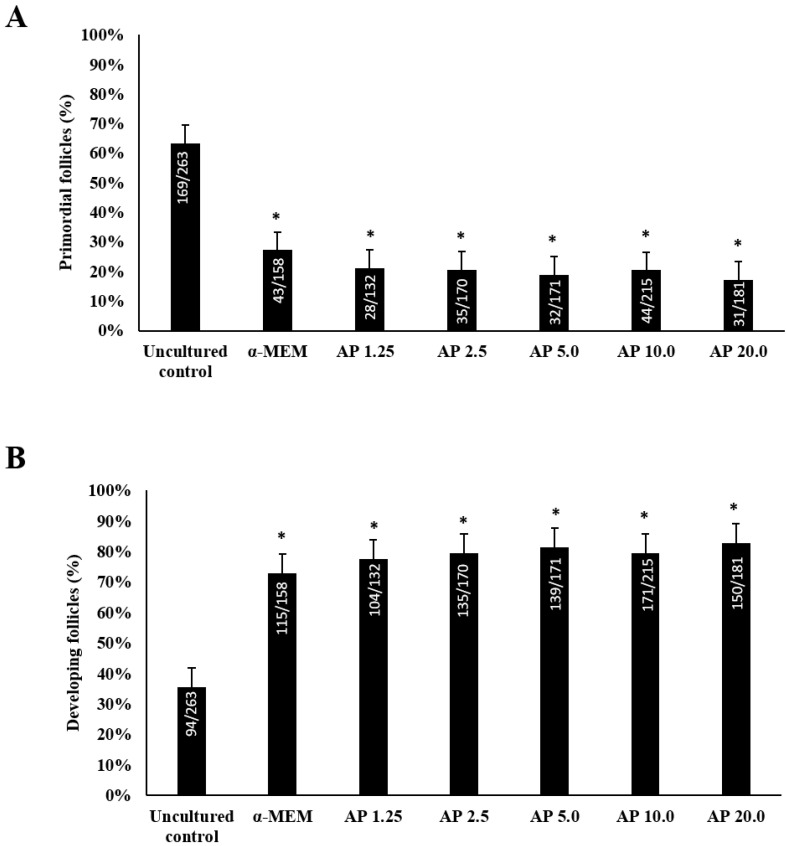
Incidence of primordial (**A**) and developing (**B**) follicles in uncultured and in 6-day cultured samples in control medium alone (α-MEM) or with 1.25, 2.5, 5.0, 10.0, or 20.0 µg/mL α-pinene (AP). * Differs significantly from uncultured tissues. Numbers of primordial and developing follicles evaluated are shown within each column.

Figure 2A shows that uncultured tissues predominantly contained primordial follicles. In contrast, after 6 days of culture, a significant increase in the rate of growing follicles (Figure 2B) was verified for all the treatments when compared with uncultured tissues (*p* < 0.05). However, the presence of α-pinene in the culture medium did not influence follicular growth.

### 3.3. Assessment of Collagen Fibers and Stromal Cell Density 

**Figure 3:**
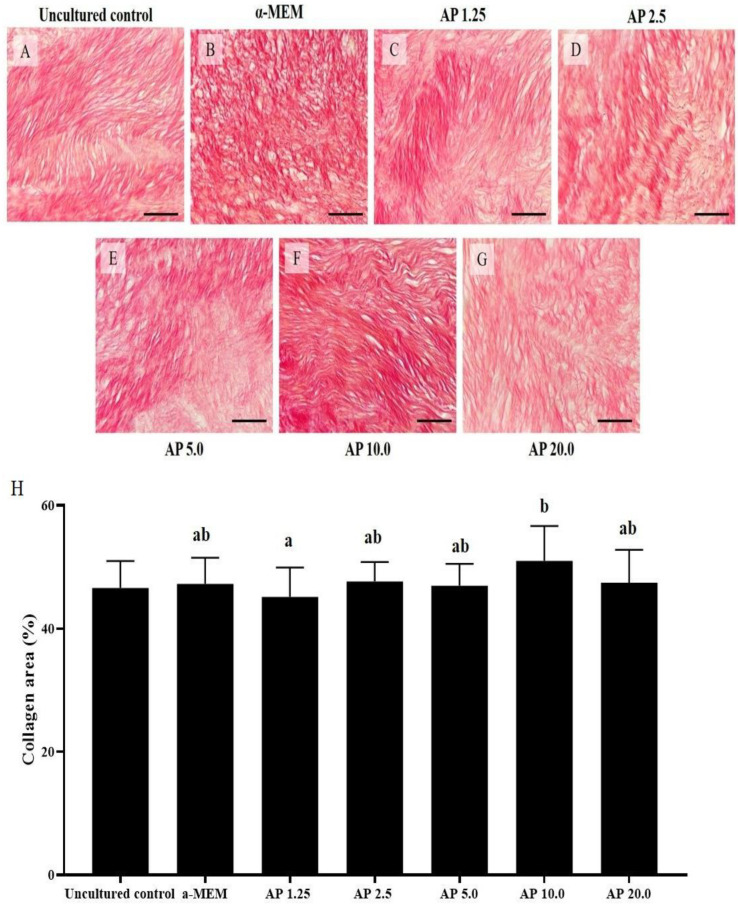
Morphology (**A**–**G**) and percentages (**H**) of collagen in uncultured samples and in samples cultured for 6 days in control medium alone (α-MEM) or with 1.25, 2.5, 5.0, 10.0, or 20.0 µg/mL α-pinene (AP). a,b: statistically significant differences between treatments. Scale bar = 100 μm.

After culturing the ovarian cortex for 6 days, α-pinene was able to maintain percentages of collagen like uncultured tissues (Figure 3). Additionally, ovarian tissues cultured with 10.0 µg/mL α-pinene had higher percentages of collagen fibers than those cultured with 1.25 µg/mL α-pinene (*p* < 0.05) but had similar percentages of collagen fibers when compared with tissues cultured in control medium (*p* > 0.05). 

**Figure 4:**
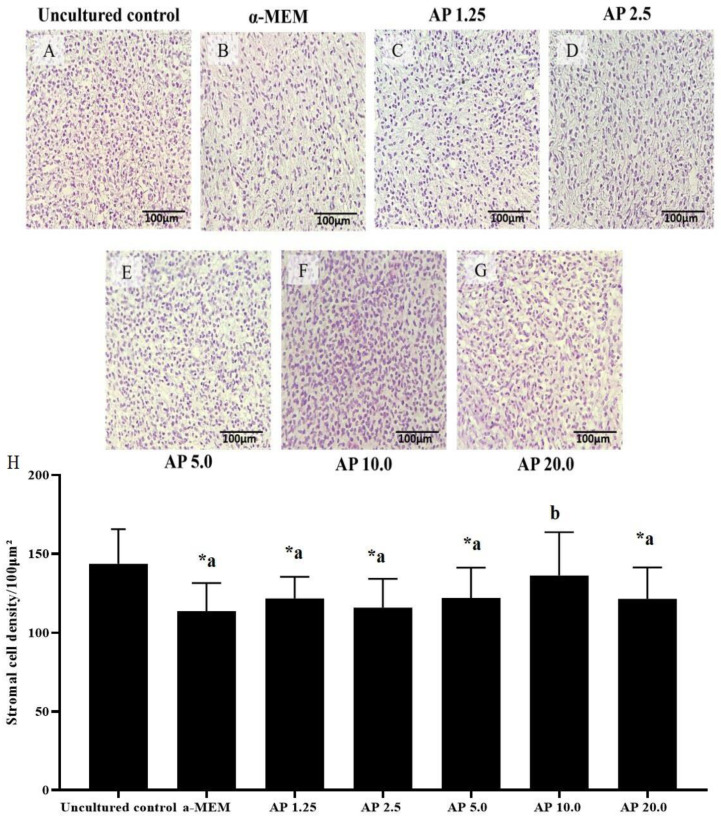
Morphology (**A**–**G**) and number (mean ± S.E.M) of stromal cells (**H**) in uncultured samples and in samples cultured for 6 days in control medium alone (α-MEM) or with 1.25, 2.5, 5.0, 10.0, or 20.0 µg/mL α-pinene (AP). a–b statistically significant differences between treatments. * Differs significantly from uncultured tissues. Scale bar = 100 μm.

**Figure 5:**
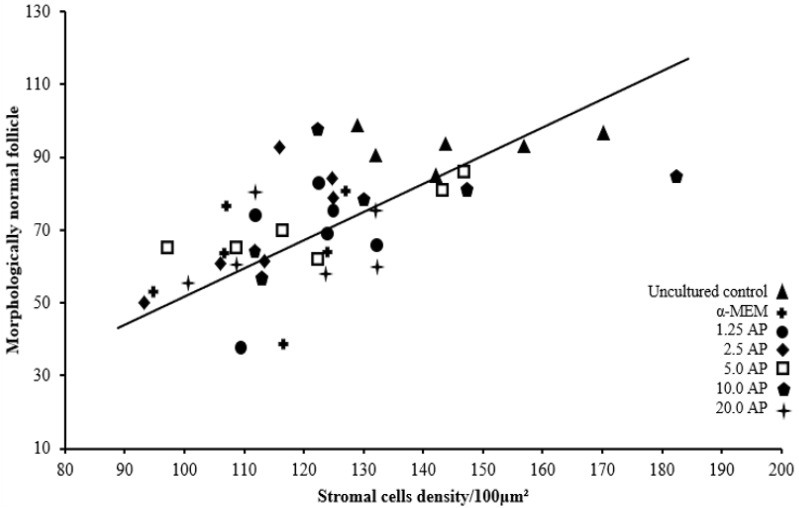
Relationships of stromal cell density with a percentage of normal follicles. The association between variables (black line) was analyzed by linear regression [Normal preantral follicles = 1.294 + (0.7450 × stromal cell density); r = +0.9833; R^2^ = 0.9669; *p* < 0.05]. Each point on the chart represents one treatment evaluated in six repetitions of the experiment.

Cultured tissues had reduced stromal cell density in all treatments, except for those cultured with 10.0 µg/mL α-pinene. Different from samples cultured in control medium, tissues cultured with 10.0 µg/mL α-pinene maintained a well-preserved ovarian structure with a density of stromal cells like those in uncultured ovarian samples (*p* < 0.05) (Figure 4). Figure 5 shows the correlation between ovarian stromal cell density and the percentage of morphologically normal follicles (*p* < 0.05). 

### 3.4. Levels of mRNA for CAT, SOD, GPX1, PRDX6 and NRF2 

**Figure 6:**
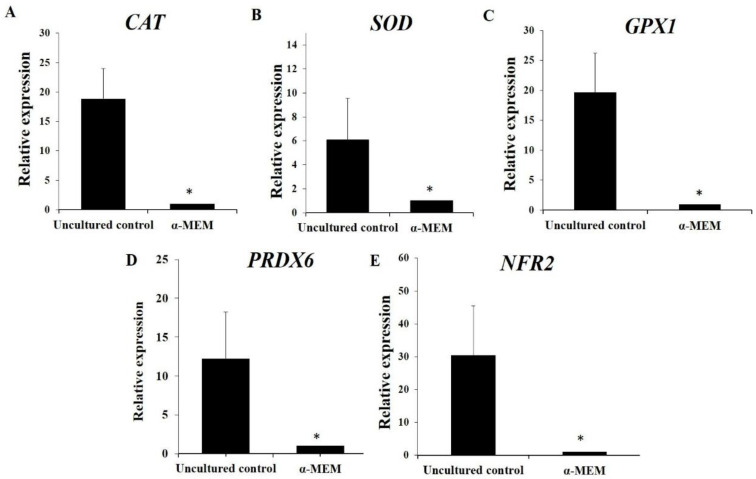
Levels of mRNA (mean ± S.E.M) for *CAT* (**A**), *SOD* (**B**), *GPX1* (**C**), *PRDX6* (**D**), and *NRF2* (**E**) in uncultured samples (*n* = 4) and in samples (*n* = 4) cultured for 6 days in control medium alone (α-MEM). * Differs significantly from uncultured tissues (*p* < 0.05).

**Figure 7:**
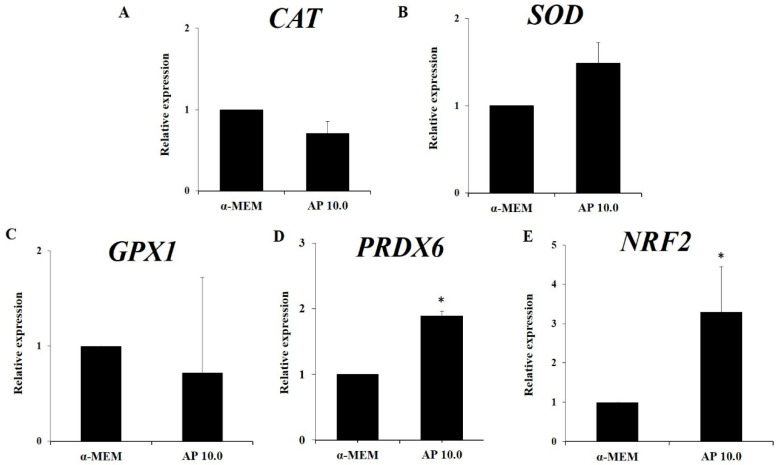
Levels of mRNA (mean ± S.E.M) for *CAT* (**A**), *SOD* (**B**), *GPX1* (**C**), *PRDX6* (**D**), and *NRF2* (**E**) in ovarian samples (*n* = 4) cultured for 6 days in control medium alone (α-MEM) or supplemented with 10.0 µg/mL α-pinene (AP). * Differs significantly from tissues cultured in control medium (*p* < 0.05).

Figure 6 shows that in vitro culture of ovarian tissues in control medium results in a significant reduction in mRNA for *NRF2*, *SOD*, *CAT*, *GPX1*, and *PRDX6*. The presence of 10.0 µg/mL α-pinene in culture medium significantly elevated the levels of *NRF2* and *PRDX6* when compared to those in samples cultured with α-MEM alone (*p* < 0.05, Figure 7). However, α-pinene did not influence the mRNA expression for *CAT*, *SOD*, and *GPX1* (*p* > 0.05). 

## 4. Discussion 

This study reports for the first time that the presence of 2.5, 5.0, or 10.0 µg/mL α-pinene in culture medium increases the rate of normal follicles after 6 days in vitro. Previously, α-pinene also increased the cell viability and expression of antioxidant enzymes in PC12 cells [19]. Bouzenna et al. [20] reported that α-pinene preserved the morphological integrity and increased cell viability of IEC-6 cells after aspirin-induced toxicity. These authors also showed that α-pinene decreased malondialdehyde levels, lipid peroxidation, and maintained the functional and structural integrity of cell membranes. α-pinene prevents lipid peroxidation by reducing the formation of thiobarbituric acid-reactive substances in human astrocytes [12]. The high lipophilic characteristic of α-pinene is an attenuator of the oxidative degradation of lipids in cell membranes [21]. In our study, α-pinene increased the expression of *PRDX6* mRNA. The modulation of this enzyme constitutes a key target to counteract the ROS-mediated lipid peroxidation that may have contributed to maintaining cell viability.

The α-pinene (10.0 µg/mL) increased stromal cell number in cultured bovine ovarian cortex. A positive relationship between morphology and stromal cell density was also observed. The ovarian stromal cells produce peptides and growth factors that support follicular development [2]. These cells play various key roles, such as multiplying and differentiating into outer myofibroblasts responsible for secreting ECM proteins, such as collagen [22]. 

α-pinene did not influence collagen fibers in the ECM of cultured ovarian tissues. This ovarian microenvironment may have a significant impact on follicle and oocyte quality [23]. Metalloproteinases (MMPs) are enzymes capable of degrading a variety of ECM proteins [24]. In a previous study, it was observed that treatment with α-pinene significantly reduced the levels of mRNA for *MMP-13*, *MMP-9*, and *MMP-2* [25]. Additionally, MMPs can be activated by reactive oxygen species (ROS) [26]. Therefore, the antioxidant capacity of α-pinene may strongly contribute to the reduction of ROS [27], allowing the ovarian tissue to maintain adequate levels of collagen during the culture period, which may have contributed to increasing the percentage of normal follicles and to maintaining the density of the stromal cells.

In vitro culture of ovarian tissues in control medium results in a significant reduction in the expression of mRNA for *NRF2*, *SOD*, *CAT*, *GPX1*, and *PRDX6*. Similar results were also reported recently by Silva et al. [7]. An imbalance in oxidant/antioxidant signaling due to an increase in supraphysiological ROS levels was also reported in in vitro cultured oocytes and embryos [28]. During in vitro culture, follicles and tissues are maintained in an environment that is different from in vivo conditions and, consequently, require adequate conditions to survive and develop [29]. The lack of antioxidant defense mechanisms elevates the levels of cellular oxygen and consequently increases ROS production, which causes homeostatic imbalance and oxidative damage to biomolecules, including nucleic acids, lipids, proteins, and carbohydrates [30]. These events cause damage to mitochondria and DNA and lipid peroxidation, which disrupts the cell membranes and ECM homeostasis. It also influences the communication between stroma, follicle, and oocyte and consequently changes gene expression in in vitro cultured follicles [31]. 

α-pinene (10.0 µg/mL) increased mRNA levels for *NRF2* and *PRDX6* in cultured tissues. Previous studies showed that α-pinene has an effective antioxidant effect through the nuclear *NRF2* factor and the increase of antioxidant enzymes [12,13]. *NRF2* is a cytoplasmic protein translocated to the nuclei by oxidative stress and is involved in cellular adaptation under oxidative challenges, as well as promoting cell survival and reducing apoptosis [32]. The *PRDX6* enzyme is a target for *NRF2*-mediated transcription in response to oxidant substances [33]. This enzyme uses phospholipid hydroperoxides as substrates and have phospholipase A2 and lysophosphatidylcholine acyltransferase activities [34]. The *PRDX6* is present in almost all organelles that produce ROS, such as lysosomes, mitochondria, and the endoplasmic reticulum, which maintain ROS homeostasis and the morphological integrity of cells and tissues [35,36]. In our study, increased levels of transcripts for *NRF2* and *PRDX6* can be linked with higher cell and stromal cell density in cultured ovarian samples. Leyens et al. [37] reported that peroxidase enzymes are involved in antioxidant defense and intracellular signaling through their activities of alkyl and hydrogen peroxide reductase in cells and tissues. In this way, α-pinene can affect both ovarian follicles and stromal cells. Additionally, the activity of these enzymes can be influenced by other factors, such as post-translational modifications and enzyme cofactor availability [38]. Thus, protein-level analysis and activity assays will provide a more comprehensive understanding of how α-pinene influences follicular survival in cultured ovarian tissues. 

## 5. Conclusions

α-pinene (10.0 µg/mL) improves follicular survival, preserves stromal cell density and collagen levels in the ECM, and increases the expression of *NRF2* and *PRDX6* mRNA after in vitro culture of ovarian tissues. The in vitro culture of ovarian tissues, however, reduces the expression of mRNA for *NRF2*, *SOD*, *CAT*, *GPX1,* and *PRDX6*.

The beneficial effects of α-pinene on follicle survival, tissue integrity, and antioxidant defense mechanisms in cultured ovarian tissues provide valuable insights into potential strategies for improving the in vitro growth of ovarian follicles in both animals and humans. The upregulation of *PRDX6* and *NRF2* indicates the role of α-pinene in enhancing the antioxidant defense mechanism within ovarian tissues. Understanding how α-pinene modulates gene expression is important for optimizing in vitro culture conditions. 

## Figures and Tables

**Table 1:** Primer pairs used for real-time PCR.

Gene	Primer Sequence (5′→3′)	Sense (S) Anti-Sense (As)	GenBank Accession No.
*GAPDH*	TGTTTGTGATGGGCGTGAACCAATG GCGCGTGGACAGTGGTCATAA	S AS	GI: 402744670
*PRDX6*	GCACCTCCTCTTACTTCCCGGA TGCGGCCGATGGTAGTAT	S AS	GI: 59858298
*GPX1*	AACGTAGCATCGCTCTGAGG GATGCCCAAACTGGTTGCAG	S AS	GI: 156602645
*SOD*	GTGAACAACCTCAACGTCGC GGGTTCTCCACCACCGTTAG	S AS	GI: 31341527
*CAT*	AAGTTCTGCATCGCCACTCA GGGGCCCTACTGTCAGACTA	S AS	GI: 402693375
*NRF2*	GACCCAGTCCAACCTTTGTC GACCCGGACTTACAGGTACT	S AS	GI: 0304941

## Data Availability

The data presented in this study can be obtained by contacting venancianunes@gmail.com.
